# Handheld Ultrasound: Technical Review and International Expert Consensus Recommendations

**DOI:** 10.3390/diagnostics16193215

**Published:** 2026-10-04

**Authors:** Christoph F. Dietrich, Michael Blaivas, Vito Cantisani, Xinwu Cui, Yi Dong, Ahmed El Kaffas, Adrian Goudie, Ole Graumann, Moritz Herzog, Thomas Karlas, Daniel Merkel, Kathleen Möller, Michael Bachman Nielsen, Christian Pállson Nolsoe, Peter J. Snelling, Daniel Wastl, Christian Jenssen

**Affiliations:** 1 Center of Education and Excellence, 3626 Thun, Switzerland; c.f.dietrich@googlemail.com; 2 Department of Internal Medicine, University of South Carolina School of Medicine, Columbia, SC 29225, USA; mike@blaivas.org; 3 Dipartimento Interdisciplinare di Benessere, Salute e Sostenibilità Ambientale, (BeSSA) Department, Sapienza University of Rome, 00161 Rome, Italy; vito.cantisani@uniroma1.it; 4 Department of Medical Ultrasound, Tongji Hospital, Tongji Medical College, Huazhong University of Science and Technology, Wuhan 430030, China; cuixinwu@live.cn; 5 State Key Laboratory for Diagnosis and Treatment of Severe Zoonotic Infectious Diseases, Tongji Hospital, Tongji Medical College, Huazhong University of Science and Technology, Wuhan 430030, China; 6 Department of Ultrasound, Xinhua Hospital Affiliated to Shanghai Jiaotong University School of Medicine, Shanghai 200082, China; drdaisydong@hotmail.com; 7 Departments of Radiology and Surgery, Moores Cancer Center, University of California San Diego, La Jolla, CA 92037, USA; aelkaffas@health.ucsd.edu; 8 Fiona Stanley Hospital, Perth, WA 6150, Australia; adrian.goudie@health.wa.gov.au; 9 Department of Radiology, Aarhus University Hospital, 8200 Aarhus, Denmark; olegraumann@dadlnet.dk; 10 Else Kröner Fresenius Center for Digital Health, Faculty of Medicine Carl Gustav Carus, Technical University Dresden, 01307 Dresden, Germany; moritz.herzog@tu-dresden.de; 11 Division of Gastroenterology, Department of Medicine II, Leipzig University Medical Center, Liebigstrasse 20, 04103 Leipzig, Germany; thomas.karlas@medizin.uni-leipzig.de; 12 Department of Internal Medicine, Caritas-Klinik Dominikus, 13467 Berlin, Germany; daniel.merkel@mhb-fontane.de; 13 Brandenburg Institute for Clinical Ultrasound (BICUS), Brandenburg Medical University, 16816 Neuruppin, Germany; 14 Medical Department I/Gastroenterology, Sana Hospital Lichtenberg, 10365 Berlin, Germany; k.moeller@live.de; 15 Department of Radiology, Rigshospitalet, Blegdamsvej 9, 2100 Copenhagen, Denmark; mbn@dadlnet.dk; 16 Center for Surgical Ultrasound, Department of Surgery, Zealand University Hospital, 4600 Køge, Denmark; cnolsoe@cnolsoe.dk; 17 Institute for Clinical Medicine, University of Copenhagen, 2400 Copenhagen, Denmark; 18 Emergency Department, Gold Coast University Hospital, Southport, QLD 4215, Australia; peter.j.snelling@gmail.com; 19 Center for Intensive Care, Hochtaunus-Kliniken Bad Homburg, 61352 Bad Homburg, Germany; daniel-wastl@t-online.de; 20 Department of Internal Medicine, Krankenhaus Märkisch Oderland, 15344 Strausberg, Germany

**Keywords:** point of care, point-of-care ultrasound, ultrasound, handheld ultrasound, guideline, regulation, policy

## Abstract

**Background**: Handheld ultrasound (HHUS) is increasingly used in point-of-care imaging because of its portability, accessibility, versatility, and comparatively low cost. **Methods**: This structured narrative technical review evaluated current HHUS technology, image quality and functionality, clinical applications, regulatory considerations, and future perspectives using recent literature, comparative studies, technical sources, and previous EFSUMB/WFUMB position papers. A structured SWOT analysis informed draft recommendations, which underwent three rounds of scientific discussion and revision by a 17-member international multidisciplinary expert panel. Each of the seven final recommendations was subsequently voted on separately, with consensus predefined as ≥80% agreement. **Results**: While HHUS devices are increasingly suitable for routine clinical use, further standardization, clinician training, and regulatory alignment are essential for widespread adoption. **Conclusions**: HHUS can expand access to focused ultrasound imaging, particularly in point-of-care and resource-limited settings. Its appropriate use requires task-specific device selection, adequate training, quality assurance, and recognition of its limitations compared with comprehensive ultrasound systems.

## 1. Introduction

The development of mobile ultrasound (US) diagnostics began as early as the 1970s, with the intention of bringing US to the patient [1,2,3,4]. The advent of portable scanners in the 1970s marked the beginning of clinician-performed US, which evolved into what is now known as point-of-care ultrasound (POCUS), encapsulating the concept of “ultrasound performed and interpreted at the bedside by the clinician” [2,3,5]. However, while these portable machines were more mobile than prior ones, many were still cart-based systems, and some were large ‘laptop’ devices that remained cumbersome. The first wireless handheld ultrasound device (HHUSD; dimension: 26 × 16 × 16 cm; weight: 1.5 kg) was the ‘MiniVisor’, developed in 1975 and distributed by OrganonTeknika in 1978 and 1979. It was primarily used for acute echocardiography (‘ultrasound cardioscope’), obstetric scanning (fetal biparietal diameter), and urinary bladder scanning for the determination of residual urine volume [6,7]. Highly portable US systems and HHUSDs, akin to a ‘visual’ stethoscope, are well suited to POCUS as they can improve patient evaluation and care by incorporating ‘insonation’ as the 5th pillar of the clinical examination alongside inspection, palpation, percussion, and auscultation [3,8,9,10].

US is now a widely available, affordable, versatile, non-invasive imaging modality that can facilitate increased patient contact, when clinicians apply POCUS. The clinician can use clinical information to perform a targeted bedside US examination, which does not necessarily replace comprehensive US imaging, but can enable effective, timely, and cost-effective clinical decisions. Given the rapid technological advances in digitization and miniaturization over the last decade, clinicians can now use pocket-sized HHUSDs at the bedside [3,10]. This development has contributed to the concept of the “echoscope” [5,11,12,13,14] and was reflected in position papers issued by the World Federation for Ultrasound in Medicine and Biology (WFUMB, 2017) [15], the European Federation of Societies for Ultrasound in Medicine and Biology (EFSUMB, 2019) [16], and the European Society of Radiology (ESR, 2019) [17]. Since then, the HHUS landscape has changed substantially. Relevant developments include semiconductor-based transducer technologies, improvements in Doppler and image quality, increasing integration of AI-assisted image acquisition and interpretation, telemedicine and teleguidance, evolving cybersecurity and data-integration requirements, and a rapidly changing commercial device market. These developments justify an updated technical review and renewed expert recommendations [3,18,19].

The objective of this review is to provide a comprehensive update of HHUSDs, in conjunction with a structured analysis of strengths, weaknesses, opportunities, and threats (SWOT) [20,21], in order to develop consensus-based statements and recommendations.

## 2. Methods

**Figure 1:**
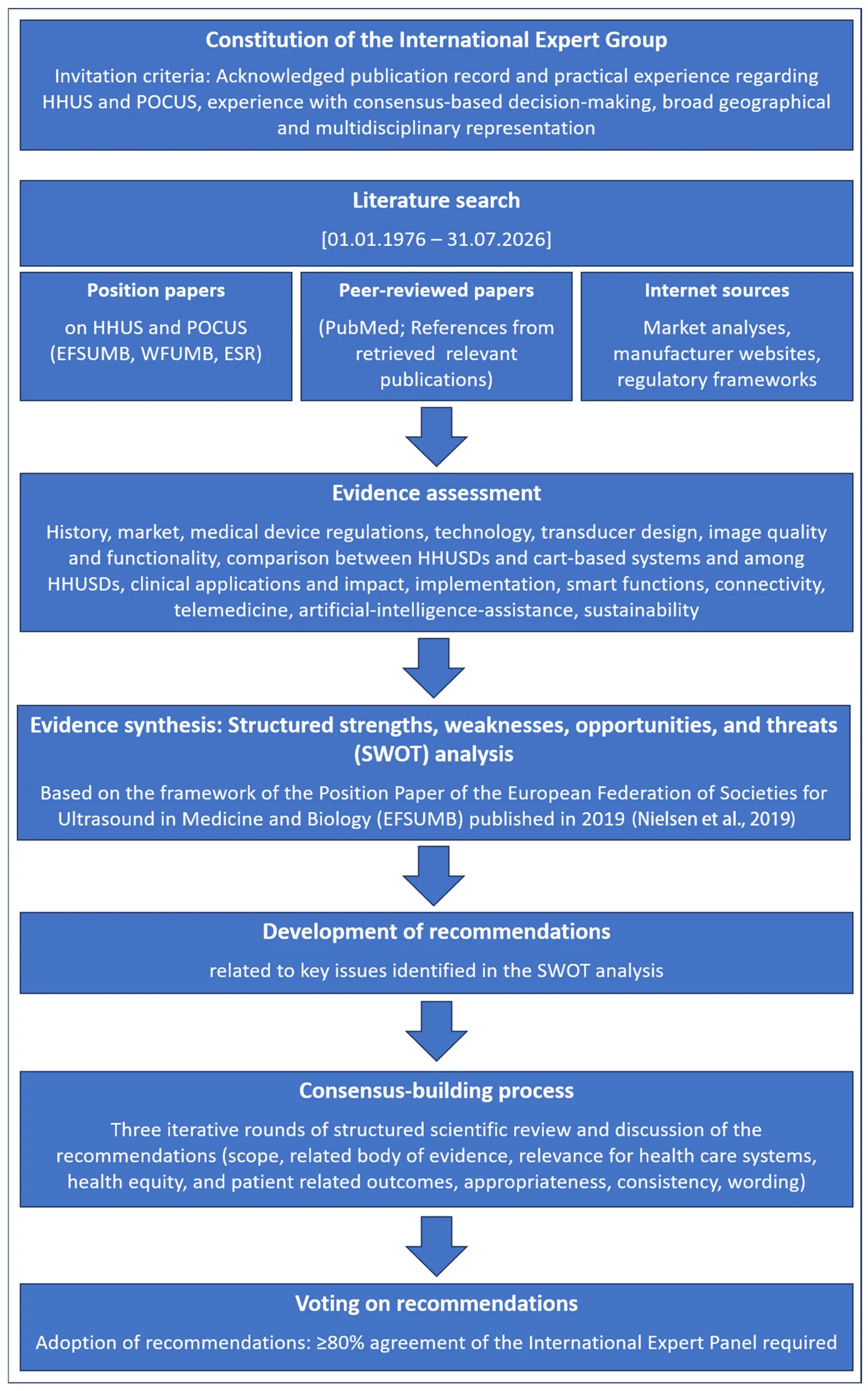
Flowchart summarizing the sequence from expert-panel constitution and evidence identification and assessment through SWOT analysis, development of recommendations, consensus-building, and final approval of the recommendations [16].

This work was designed as a structured narrative technical review combined with an international expert consensus process; it was not designed as a systematic review or a Delphi study. The review provides an update on handheld ultrasound devices (HHUSDs), with emphasis on current technological capabilities, image quality and functionality, clinical applications, market developments, regulatory considerations, artificial intelligence (AI), telemedicine, cybersecurity, and future perspectives. The evidence identified in the literature and technical sources was interpreted together with a structured strengths, weaknesses, opportunities, and threats (SWOT) analysis to support the formulation of consensus recommendations [20,21] (Figure 1).

The literature search was based primarily on PubMed using the search string (hand-held[tiab] OR handheld[tiab] OR hand-carried[tiab] OR pocket-sized[tiab] OR pocket-size[tiab]) AND (ultrasound[tiab] OR ultrasonography[tiab] OR ultrasonic[tiab]) [1 January 1976–31 July 2026] without formal language restriction. No formal systematic-review protocol was registered. After screening the titles and abstracts, publications were considered when they were relevant to HHUS technology, transducer design, image quality or technical performance, comparisons with conventional or cart-based ultrasound, clinical applications, implementation, regulation, connectivity and smart functions, AI, telemedicine, sustainability, and/or recommendations concerning HHUS/POCUS. The PubMed search was supplemented by relevant references identified from included publications and by previous EFSUMB, WFUMB, and ESR position papers [5,15,16,17].

For device-specific technical characteristics that were incompletely reported in peer-reviewed publications, publicly available manufacturer websites, technical data sheets, and product information were additionally reviewed. HHUSD manufacturers were identified based on information from current market analyses [18,19], comparative studies [22,23,24,25,26,27,28,29], and an internet search (Google Chrome, v2026; last search: 31 July 2026) using the search terms “handheld ultrasound”, “pocket-sized ultrasound”, “Portable ultrasound”, and “wireless ultrasound”. Only English-language manufacturer websites were evaluated that contained precise technical specifications, particularly regarding transducer configuration and technology, available ultrasound modes, operating system, connectivity, weight, battery life, charging time, AI technology, and additional accessories. Information from 23 manufacturers identified during preparation of the technical review was used to characterize representative commercially available HHUS technologies rather than to establish a complete global commercial registry. Because device availability, regulatory approval, software functions, and technical specifications vary geographically and change rapidly, these data should be regarded as illustrative and time-dependent rather than as a comprehensive inventory of all commercially available HHUSDs.

Comparative studies of HHUSDs and conventional or cart-based ultrasound systems were assessed with particular attention to the clinical task, diagnostic adequacy, image quality, technical performance, usability, and device-specific limitations. Evidence directly evaluating image quality or technical performance was distinguished, where possible, from studies in which diagnostic performance or clinical utility provided indirect evidence of imaging adequacy. The review did not apply formal certainty-of-evidence grading because its purpose was a broad technical and clinical narrative synthesis rather than a systematic evidence assessment.

The International Expert Panel comprised 17 experts. Panel members were selected on the basis of internationally recognized expertise, publication record, and previous contributions to HHUS, POCUS, ultrasound technology, guideline or consensus development, or related scientific work. Broad geographical and multidisciplinary representation was intentionally sought. The panel included experts from Europe, North America, Asia, and Australia and represented gastroenterology, radiology, emergency medicine, intensive care medicine, and ultrasound technology. Seventeen experts were invited and all agreed to participate.

After completion of the literature review and SWOT analysis, the first and last authors (C.F.D. and C.J.) prepared the initial draft recommendations on the basis of the evidence summarized in the manuscript and informed by previous EFSUMB and WFUMB position papers. The complete manuscript and proposed recommendations were circulated electronically to all panel members. The manuscript and recommendations then underwent three iterative rounds of structured scientific review and discussion by e-mail. During these rounds, the wording, scope, rationale, and supporting evidence were revised in response to panel comments. Differences of opinion were addressed through discussion, clarification of the underlying evidence, and incorporation of amendments until no further substantive scientific or methodological objections remained. These discussion rounds were not voting rounds and did not constitute a formal or modified Delphi process.

For each recommendation, panel members were asked whether they agreed with the final proposed wording. Each recommendation was voted on separately. Consensus was predefined as agreement by at least 80% of participating panel members, with definitive rejection of a proposed recommendation if agreement was 50% or below. For agreement levels above 50% and below 80%, a nominal group process to develop alternative wording was planned, whereby the alternative proposal would be definitively rejected if it failed to reach at least 80% agreement. All 17 panel members provided a response for each of the seven recommendations (100% response rate); no abstentions occurred, and all seven recommendations achieved unanimous agreement (17/17; 100%). Therefore, no recommendation required further revision or repeat voting. All relevant conflicts of interest were disclosed as reported in the manuscript. No additional formal conflict-of-interest restrictions or exclusions were applied during the expert discussion or voting process. The recommendations address general principles of HHUS technology, quality, training, implementation, and clinical use rather than endorsement of specific commercial devices or manufacturers. Device-specific information was used descriptively, and all panel members participated in the discussion and final voting.

## 3. Results

### 3.1. SWOT Analysis of HHUSDs

A SWOT analysis identifies the essential internal and external factors that influence the strategies for achieving a certain goal [20,21]. The SWOT analysis in Table 1 represents the international expert group’s consensus-based interpretation of the available literature rather than a formal systematic evidence synthesis.

### 3.2. Market Growth and Increase in Peer-Reviewed Publications

There are now approximately 100 handheld ultrasound devices available worldwide, offered by both established ultrasound manufacturers and an increasing number of specialized technology companies. Market analyses consistently predict continued expansion of the HHUSD market over the coming years at compound annual growth rates of up to 13.5%, reflecting increasing clinical adoption and ongoing technological innovation. Current analyses suggest particularly strong growth in North America and the Asia-Pacific region.

The increasing adoption of HHUSDs is driven by advances in miniaturization, wireless technology, and artificial intelligence, together with growing demand for point-of-care diagnostics, telemedicine, and rapid bedside imaging across a broad range of clinical settings [18,19].

Beyond commercial growth, the increasing incorporation of HHUSDs into emergency medicine, intensive care, primary care, medical education, and global health programs illustrates their growing clinical acceptance and practical impact. This is illustrated by the fact that the volume of PubMed-indexed literature related to HHUS has increased substantially over the past three decades. Using the search strategy described in the Methods, cumulative publication counts increased from 84 articles in 1995 to 300 in 2005, 803 in 2015, 2124 in 2025, and 2285 by the end of the literature search on 31 July 2026, illustrating the marked growth of scientific interest in handheld ultrasound.

### 3.3. Medical Device Regulations

In some, but not all, locations aftermarket screens can be deemed ‘not for medical use’, which could create a potential dilemma for the user regarding medical regulation and for the vendor regarding software compatibility. There remains uncertainty regarding the legalities and conformity of these device combinations with regulations [30,31].

The business model of the majority of HHUSD suppliers (specifically the vendors) is to provide the HHUSD with the associated application software purchased from the HHUSD supplier, which is then installed on an aftermarket smart device that serves as the screen. This can create theoretical issues from a regulatory standpoint. Only a minority of manufacturers, particularly those offering HHUSDs with a cable connection between the transducer and screen, ensure certification of the entire system as a medical device.

Nevertheless, given the device’s intended purposes and its use in clinical settings, approvals for medical device use can be obtained. Given that many end devices communicate with the internet, there remains a theoretical risk of data being breached.

### 3.4. Technical Characteristics

#### 3.4.1. Transducers

##### Array Technology

Currently, the majority of HHUSDs on the market are still equipped with conventional polycrystalline piezoelectric ceramic (PZT) arrays. In contrast to high-end cart-based systems, single crystal transducers have so far been little represented on the HHUSD market. In the technical data sheets or product descriptions from the websites of a total of 23 manufacturers of HHUSDs, we were only able to identify two recently launched systems that are equipped with single crystal transducers.

Compared with conventional PZT ceramic arrays, single-crystal transducers have a higher bandwidth and a more efficient electromechanical coupling, thereby offering higher spatial resolution, deeper penetration, and enhanced signal-to-noise ratio. These factors result in higher image quality and enhanced Doppler and harmonic imaging capabilities, which would be advantageous for HHUSDs, while the higher costs are a relevant disadvantage [32].

Transducer technologies have undergone a significant advancement with the development of the first semiconductor-based transducers. Compared to conventional piezoelectric systems, these technologies offer improved bandwidth and reduced manufacturing costs due to the scaling effects of the semiconductor industry. Furthermore, the required electronic processing can be integrated directly into the same wafer, enabling miniaturization and enhanced energy efficiency. To date, two main semiconductor transducer technologies have been translated into clinical applications and are used in HHUSDs, cMUT (capacitive Micromachined Ultrasonic Transducer) and pMUT (piezoelectric Micromachined Ultrasonic Transducer) [33].

The only clinically available HHUSDs using cMUT technology are the three development stages of the BUTTERFLY iQ (iQ, iQ+, iQ3) from Butterfly Network Inc. (Burlington, MA, USA) [34]. Although cMUTs provide high bandwidth and sensitivity [35], their transmit capabilities at the current state of technology are inherently limited by a deficit in output surface pressure compared to piezoelectric array transducers, resulting in a loss in penetration and sensitivity to detect blood flow, and the inherently nonlinear response of cMUTs, hampering their ability to perform harmonic imaging [34,36,37,38,39]. Piezoelectric micromachined ultrasonic transducers (pMUTs) represent an alternative approach, utilizing thin piezo-active membranes integrated into silicon substrates. In contrast to cMUTs, pMUTs offer enhanced transmit performance but exhibit reduced sensitivity in receive mode [40]. First-generation probes based on this technology are now commercially available, including one commercially available pMUT-HHUSD (Exo Iris; Santa Clara, CA, USA) [33]. Independent of transducer material, operating frequency and bandwidth are major determinants of ultrasound image characteristics. Higher frequencies generally improve spatial resolution but reduce penetration depth because of increased acoustic attenuation, whereas lower frequencies provide greater penetration at the expense of spatial resolution. Consequently, the clinically appropriate frequency range depends on the target depth and application, with higher frequencies typically used for superficial structures and lower frequencies for abdominal and other deeper targets. The effective imaging performance of an HHUSD therefore cannot be inferred from transducer technology alone but depends on the interaction between operating frequency, bandwidth, aperture, beamforming, signal processing, and the clinical task.

Most conventional handheld probes use one-dimensional (1D) arrays, in which elements are arranged along a single axis and electronic beam steering and focusing are primarily performed within the imaging plane. Two-dimensional (2D) or matrix arrays contain elements distributed in two dimensions and can enable electronic focusing and steering in both azimuthal and elevational directions, thereby facilitating volumetric or three-dimensional imaging as well as improving image quality at greater depths and in obese patients. However, matrix arrays require substantially more elements, channels, data processing, and power, which remain relevant constraints for highly miniaturized handheld systems. Semiconductor-based cMUT and pMUT technologies may facilitate future integration of increasingly complex array architectures because transducer elements and electronics can be manufactured and integrated using semiconductor processes [34,39,40,41,42].

##### Transducer Types and Geometry

Recent advancements in both transducer fabrication and chip design have facilitated a growing diversity of transducer geometries in HHUSDs. Some vendors offer up to seven HHUSDs with conventional piezoelectric linear arrays, convex and microconvex arrays, phased arrays, and intracavitary probes for specific fields of application (e.g., Clarius, Vancouver, BC, Canada; Asus, Taipei, Taiwan; Youkey, Wuhan, China). Microconvex (microcurvilinear) arrays are convex arrays with a smaller footprint and aperture and a tighter radius of curvature than conventional abdominal convex arrays. Their smaller footprint facilitates scanning through restricted acoustic windows while retaining a widening field of view with increasing depth.

An alternative strategy is to offer a “whole body” or “all-in-one” approach with one single HHUSD. There are various technological solutions for this approach. An increasing number of devices on the market follow the “dual head” approach. “Dual head” HHUSDs incorporate two different arrays at the opposite ends of the probe, combining a linear array with a convex array or a phased array. A further development is the concept of the “dual head-3 array” or “3-in-1” scanner. These systems combine a linear and a convex transducer, with the former electronically switchable to a virtual phased-array function. Even greater flexibility is achieved with modular systems that offer exchangeable transducer arrays, allowing users to switch arrays and applications on a single handheld device (“plug and play” concept, e.g., Prunus, Shenzhen, China; Youkey). The need for physical switching is further mitigated in systems employing advanced beamforming, capable of emulating different transducer geometries through a single array. Here especially transducer types that can be integrated with chips, like pMUT or cMUT, have an advantage.

Modern HHUSDs weigh between 140 and 307 g. They are increasingly manufactured to meet military-grade durability standards (MIL-STD-810G), including resistance to impacts and drops, waterproofing up to 1–1.5 m (IP67/68), and compatibility with disinfection or, in some cases, sterilization procedures. These devices connect to portable monitors either via cable and/or wireless technologies such as Bluetooth or Wi-Fi, offering a trade-off between greater portability and higher data transfer rates.

#### 3.4.2. Screen and System Connectivity

In some HHUSDs, the US probe is offered in combination with a Conformité Européenne (CE)-certified and wire-connected screen or tablet (“closed system”) (e.g., Lumify, Philips Healthcare, Eindhoven, Netherlands; Kosmos, EchoNous, Washington DC, USA). This has the advantage that the screen and probe are compatible, the entire system is approved as a medical device, and the systems are used exclusively for medical diagnostic purposes. The disadvantages include higher CE certification costs, higher acquisition prices for small-scale tablet or phone purchases by the vendor, and rapid screen obsolescence, which can occur many years before product release. Most vendors rely on a connection to a commercially available, non-certified medical device (e.g., a smartphone or tablet) (“open systems”). This has the advantage that all users can use their own mobile devices, which they already carry for official or private use, and with which they are well acquainted. In these cases, most vendors will have a list of approved devices and models, with tested functionality across the most common operating systems (e.g., Android, Windows, and iOS). Not all HHUSD vendors offer apps and connectivity across all mobile operating systems.

The lack of strict separation of the storage and transfer of patient data from private applications is problematic when US devices are coupled to personal tablets or smartphones, leading some facilities to use locked tablets or smartphones that provide greater security. Furthermore, the risk of a data leak or breach, or of the diagnostic US software application malfunctioning due to malware, is greater than with closed systems.

For smartphone-based open systems, the use of dedicated institutional smartphones or tablets is preferable whenever feasible. Where personal mobile devices are used, secure managed institutional user profiles or restricted guest-user accounts should be employed to separate professional and private data. Additional safeguards should include encrypted local storage and data transfer, multi-factor authentication, automatic logout, role-based access control, anonymized or pseudonymized patient identifiers where appropriate, regular software updates, and integration with institutional mobile device management systems. These measures help minimize cybersecurity risks, prevent inadvertent disclosure of patient information, and facilitate compliance with national and international data protection regulations.

Although not mandatory in all countries, it is essential for reasons of comparability with other imaging findings and in follow-up, for quality control, competency assessment, and for demonstration in interdisciplinary dialogues essential to be able to store US images in the medical unit’s digital documentation systems, including Picture Archiving and Communication System (PACS), Electronic Health Records (EHRs), and other systems [43]. Moreover, findings should be reproducible to ensure diagnoses are reproducible across doctors and, if necessary, legal entities. Particularly when less experienced US practitioners use HHUSDs during POCUS examinations and US training, findings must be reviewed and supervised by experienced US practitioners. Storage of patient data (patient identification, images/video files, reports) must be protected against unauthorized access while allowing authorized access for supervising experts or for consultations. All vendors offer structured storage for patient data, image and video files, and, in some cases, reports on the connected smart device via their app. Several vendors provide external cloud storage. DICOM transfer, particularly connectivity to PACS and EHR systems, and, sometimes, automated billing, are currently offered only by a few providers.

In most cases, advanced data storage and transfer options must be purchased separately. However, external cloud storage poses a risk of data theft and misuse that is difficult to control. Transferring data from a consumer smart device to a PACS or EHR exposes a doctor’s office, hospital, or other medical facility to the risk of a cyberattack. Some vendors now offer multi-factor authentication to increase patient data security.

Hygiene issues should not be overlooked when monitors or tablets used with patients are subsequently used in private settings. Separation should be ensured in compliance with strict hygiene regulations. Unfortunately, disinfectants can permanently damage the devices’ housings. Products that can withstand disinfectants should be labeled explicitly so that they can be safely reprocessed after use on infectious patients.

HHUSDs are by nature small, portable systems that require mobile devices with correspondingly small displays. Advantages of a smaller terminal device include practical handling, easy storage, and device transportation in the professional environment. Screen size limitations may be associated with a loss of image information compared with traditional US systems, but did not appear to affect interpretation quality in one study [44]. A smaller screen could also hinder labeling images, though voice recognition software is rapidly improving and is now offered by some HHUSD manufacturers. The use of virtual reality glasses instead of smartphones or tablets can potentially improve image quality while eliminating the need for optimal screen positioning and allowing the patient to be viewed at the same time, which offers invaluable advantages, especially when working in confined spaces and for PoCUS interventions, remote supervision, teleguidance and training [45,46,47,48,49]. Another new approach is the use of HHUSDs which can be connected to more advanced portable US systems of the same vendor as a wireless additional transducer (e.g., Mindray, Shenzhen, China, and GE HealthCare, Chicago, IL, USA). This combines the advantages of a mobile, wireless, completely disinfectable US probe with easier workflow, high-definition screens of ≥19 inches, the full range of AI support and direct data transfer to and from the PACS and or hospital information system provided by the advanced US system.

#### 3.4.3. Supplementary Devices

Some HHUSDs offer supplementary devices, including protective cases, wireless charging pads, and tablet or mobile phone stands. Most conventional piezoelectric HHUSDs allow a maximum scanning time of approximately 1 h. The two cMUT- and pMUT-based HHUSDs on the market have a scanning time of up to two hours. Some devices achieve longer scanning times of 3 to 5 h by drawing power from the battery of the connected smart device. Alternative options for optimizing total scanning time include swappable battery systems, an external power bank, and a hard case that supports high-speed charging (e.g., TE Air, Mindray). Only a few vendors provide dedicated units (modified smartphones like the AirSight by Mindray or tablets like the Kosmos Bridge tablet by EchoNous and the FuturePad for the Philips Lumify), which are robust, IP67-protected, and, therefore, designed for medical environments. Very few vendors (Mindray, Youkey) offer virtual reality glasses as a supplement to their HHUSDs.

### 3.5. Image Quality and Functionality

There is a wide range of HHUSD image quality, mainly due to differences in transducer technology, screen quality, and post-processing software. Ancillary US application capabilities, such as color and spectral Doppler, can vary significantly, and the industry routinely introduces new features and functions over time.

Comparing image quality between cart-based US systems and HHUSDs and among HHUSDs is difficult because devices are designed for different intended uses and performance goals.

Phantom studies can quantify individual physical parameters, including spatial resolution and grayscale or contrast discrimination [25,50,51,52,53], but these measurements do not necessarily predict the clinical adequacy of an image for a specific diagnostic question [52]. In 2024, a study that used the Edinburgh Pipe Phantom compared the Resolution Integral (R)—the ratio of ultrasound beam penetration to ultrasound beam width—across 19 HHUSDs [25]. These results were benchmarked against the R values reported for 142 cart-based systems reported by the same group in 2022 [53]. Overall, HHUSDs showed significantly inferior image performance, an effect that was more pronounced for cardiac and abdominal applications than for small-parts imaging. For all cardiac HHUSD applications, R values fell below even the lowest value recorded for cart-based systems, whereas small-parts applications showed considerable overlap between the two device categories [25]. In another study using a different ultrasound phantom to measure axial and lateral resolution in five HHUSDs and two high-end cart-based ultrasound systems, some of the HHUSDs significantly outperformed the cart-based systems on this physical metric. Notably, however, phantom-based resolution did not predict clinical performance: cart-based systems were superior at identifying real anatomical structures (layers of the sigmoid colon and portal-field substructures) in human subjects, despite the HHUSDs’ better phantom scores [52].

Objective comparison of overall B-mode image quality across clinical settings remains challenging because image quality is multidimensional and depends on factors including operating frequency, penetration depth, spatial and contrast resolution, transducer technology, image-processing algorithms, display characteristics, examination conditions, and the specific diagnostic task. Published comparative clinical studies between HHUSDs and cart-based ultrasound systems vary widely in methodology, clinical scope (focused versus broad indications), endpoints, examiner experience, device tier (portable point of care to high-end), and the relative technological generation of the cart-based comparators—some of which predate the HHUSDs being tested by several years. This heterogeneity limits the generalizability of the results of these studies.

Most comparative studies focus on clinical endpoints rather than image quality, as reflected in a systematic review and the related EFSUMB position paper from 2019 [16,54]. In a small, randomized study performed in an emergency department, diagnostic accuracy of cardiac, lung, biliary, renal, or abdominal aorta point-of-care studies using an HHUSD was similar to that obtained with a portable cart-based system [55]. Concurrently, several observational studies also demonstrated a high correlation between abdominal and lung ultrasound findings obtained using HHUSDs and conventional ultrasound systems in patients from emergency, intensive care, internal medicine and geriatric departments [11,12,54,56,57,58,59,60,61,62]. This applies in particular to relatively simple pathological conditions, e.g., the detection of free abdominal fluid, cholelithiasis/cholecystitis, cysts in parenchymal organs, abdominal aortic aneurysms, hydronephrosis, pleural effusions, B-lines, pulmonary consolidations, and inferior vena cava width [11,12,23,24,26,54,56,57,58,59,60,61,62]. A high degree of agreement between HHUSD and standard ultrasound findings has also been reported for certain gynecological findings [63], intrauterine pregnancy [64,65,66,67], pediatric thyroid findings (goiter, nodules) [68], simple musculoskeletal findings [69,70,71], intima-media thickness measurements [72], the detection of fatty liver [67], and the detection of retinal detachment [73]. For cardiac applications, a systematic review and meta-analysis of predominantly prospective studies demonstrated that experienced examiners using HHUSDs achieve diagnostic accuracy in the assessment of left ventricular structures and function comparable to that obtained with cart-based transthoracic echocardiography [74]. This is consistent also with the results of a large prospective blinded comparative study showing a high inter-device consistency of morphological and functional echocardiographic assessments between a new-generation HHUSD equipped with color, pulse-wave and tissue Doppler capabilities and a high-end cart-based ultrasound system [75]. Since some HHUSDs now also feature pulse-wave and, more recently, continuous-wave Doppler capabilities, this technology can also be used to detect and classify the severity of aortic and mitral valve disease with an accuracy comparable to standard echocardiography [76,77,78]. Recent studies have also shown that even less-experienced examiners can determine left ventricular ejection fraction using AI-assisted handheld echocardiography as accurately as cardiologists using conventional transthoracic echocardiography on high-end systems [79,80,81,82].

Limitations in diagnostic performance described in these studies were related to the detection and characterization of complex lesions of parenchymal abdominal organs, the detection of abdominal lymphadenopathy, precise measurement of the length of parenchymal abdominal organs, detection of ectopic pregnancy, assessment of superficial pathologies and deep vascular structures, and the diagnosis of posterior vitreous detachment [11,12,54,57,58,60,73,83,84,85,86], as well as the accuracy of right ventricular function assessment using handheld echocardiography [87,88]. These limitations may, at least in part, reflect technical constraints of currently available HHUSDs, including depth penetration, elevational beam width, and spatial resolution, as demonstrated in phantom studies described above [25,51,52]. This explanation is consistent with the results of a study in which a panel of experts compared image quality across several HHUSDs and a high-end ultrasound system using blinded video clips of scans of three defined abdominal regions (liver hilum, pancreas, and sigmoid colon). The high-end systems outperformed all tested HHUSDs in every expert-rated aspect of image quality [29].

In the majority of comparative studies, image quality of HHUSDs was described as reasonable or adequate in comparison to cart-based systems, although objective parameters were rarely used. Almost all studies instead report a “subjective impression” or grade B-scan image quality on a Likert scale when measuring B-scan image quality [89], as determined by the evaluating examiners [11,12,54,56,57,62,63,73,83,84,85,86,90,91,92,93,94,95].

An open question is whether all available HHUSDs are comparable in B-scan image quality and whether they are similar in handling, haptics, software, and other features. To date, only seven studies have directly compared HHUSDs from different manufacturers in the same test subjects or clinical scenarios (Table 2). These studies show that expert-rated B-scan image quality varies considerably across HHUSDs and depends heavily on the clinical application scenario.

Device handling, software functionality, image quality, and operator preference also differ substantially among systems, and no single HHUSD is consistently superior across all evaluated categories and clinical applications [22,23,24,26,27,28,29]. Device selection should therefore be indication-specific and matched to the required image quality, transducer and software capabilities, workflow, and operator expertise rather than guided by a generalized device ranking.

Whether the differences across these studies are relevant to clinical outcomes must be clarified in future studies. To investigate these questions, standardized frameworks to objectively assess the image quality of new devices are urgently needed, with different parameters proposed, but missing clinical validation [25,52,96].

### 3.6. Clinical Applications

HHUSDs are now used across a broad range of clinical settings, including emergency medicine, intensive care, anesthesiology, primary and palliative care, obstetrics, and medical education [3,4,60,65,97,98,99,100,101]. Their principal clinical advantage is immediate availability at the point of care, particularly when a focused question must be answered rapidly and the required information can be obtained with the image quality and functionality of the available device. HHUSDs have been used successfully for focused cardiac assessment in non-cardiology settings [102], assessment of fluid overload in dialysis patients [103], evaluation of systemic venous congestion using the Venous Excess Ultrasound (VExUS) score [104], pulmonary congestion using B-line assessment [105], and screening for abdominal aortic aneurysms [61,99]. During the COVID-19 pandemic, their portability and ease of isolation and disinfection were particularly advantageous for bedside cardiopulmonary assessment [106].

HHUSDs are also well suited to focused procedural guidance and urgent bedside applications, including difficult peripheral intravenous catheter placement [13,107,108], nerve blocks [109], nasogastric tube placement [110], and emergency management [111,112,113]. Beyond these procedural uses, HHUS has found application across a wide range of clinical specialties and settings—intensive care [104], geriatric medicine [57], gastroenterology [12,95,114], palliative care [90,115,116], hand surgery [69,71], sports medicine [117], disaster and military medicine [118], aviation medicine [119,120], and remote or resource-limited settings [121,122,123]—reflecting the broad portability advantage of these devices and their reliability for a wide range of clinical issues relevant to decision-making. More recently, advanced ultrasound technologies have also begun entering the realm of HHUSDs, including quantitative multiparametric methods for diagnosing hepatic steatosis and fibrosis [124,125,126]. Contrast-enhanced ultrasound, on the other hand, is not yet available with HHUSDs [127].

However, the clinical suitability of HHUS should not be equated with equivalence to conventional or high-end ultrasound for all indications. As demonstrated by the comparative studies summarized in Table 2, image quality and technical capabilities vary substantially among HHUSDs and according to the clinical application. For focused questions, HHUS may provide clinically adequate information and results comparable to conventional ultrasound, whereas examinations requiring high spatial or contrast resolution, deep penetration, advanced Doppler techniques, multiparametric assessment, or comprehensive organ evaluation may exceed the capabilities of individual handheld systems. This applies in particular to the characterization of focal solid and complex cystic lesions, the evaluation of complex organ pathologies, the assessment of tissue perfusion, the differential diagnosis of cardiac valve pathologies, and detailed prenatal diagnostics. The decision to use HHUS should therefore be based on the clinical question, required imaging modalities and quality, device capabilities, examination conditions, and operator expertise rather than assuming uniform equivalence with conventional ultrasound across all applications. If the required diagnostic information cannot be obtained with sufficient quality or confidence, comprehensive ultrasound using a conventional or high-end system, or another appropriate imaging modality, should not be delayed.

The accessibility of HHUSDs has also expanded the range of potential users. In addition to physicians, sonographers, nurses, midwives, paramedics, physical therapists, physician assistants, and medical students are increasingly being trained in focused HHUS applications [3,16,90,97,100,128,129,130,131]. HHUS may also provide a practical entry point for teaching anatomy, physiology, physical examination, and focused ultrasound skills to medical students [3,101,132,133,134,135,136,137,138,139,140,141,142,143,144]. Appropriate training, supervision, documentation, and quality assurance remain essential and should correspond to the complexity and clinical consequences of the examination.

### 3.7. Practical Issues

If several users share an HHUSD, a designated storage location or tracking technology must be available so the device does not get misplaced or lost or require searching for it. The transport and storage of the device during rounds should also be regulated, for example, with a device bag on the ward trolley or a shoulder bag for the physician. The manufacturer’s instructions for various HHUSDs, which vary greatly in terms of water resistance, disinfection options, shock and impact resistance, charging and operational time, and connectivity, should be taken into account alongside the technical parameters before purchase and clinical use.

### 3.8. Telemedical Applications

#### 3.8.1. Teleguidance and Tele-Mentoring

Thanks to their smart features, many HHUSD vendors not only offer online storage for images and video clips, but also provide remote, real-time access via apps to examinations performed using HHUSDs. This enables telemedical support, which is successfully utilized for training purposes [140] as well as for expert supervision and consultation for less experienced examiners and in remote areas [121,122,145,146,147,148,149]. In particular, the combination of tele-mentoring and AI support has been shown to rapidly improve the performance of general practitioners with limited experience in cardiological POCUS applications [150].

#### 3.8.2. Supervised Patient Self-Scanning

In principle, individuals without medical training could use HHUSDs to image themselves in a telemedical context with appropriate training and potentially AI guidance. This has been demonstrated, for example, in pilot studies for systematic thoracic scanning for B-line quantification in the context of home monitoring and therapy management of heart failure patients [151,152,153,154] and in pregnancy care [65]. In Israel, a simple HHUSD linked to a smartphone is being distributed in large numbers by a company to health care organizations to provide telemedicine-guided self-examination for pregnant women. The goal is to reduce non-indicated emergency department utilization [155,156,157]. The boundary between these approaches and medically unsupervised, potentially dangerous consumer applications, such as the now-insolvent commercial HHUSD provider “baby watcher” (Netherlands) [158], becomes blurred and possibly uncontrollable. In Germany, this practice was banned after the NISV (Ordinance on Protection against Harmful Effects of Non-Ionizing Radiation in Human Applications) came into force [159].

### 3.9. Artificial Intelligence Support

Traditionally, US has been used by experienced operators, often with years of specialized training, and by expert readers versed in interpreting data (e.g., radiologists, gastroenterologists). However, POCUS using HHUSDs is often performed by less experienced operators, and image interpretation is mainly performed by readers with limited experience. This is especially true for many proposed future clinical, rural, global, and at-home applications. The use of AI in HHUSDs has two main objectives:

1. Guidance: guiding operators in data acquisition, and
2. Interpretation: facilitating the interpretation of acquired data.

Although the two underlying limitations—namely, uncertainties regarding accurate image acquisition and interpretation problems—are generally relevant to conventional US, they are especially critical in POCUS due to the limited experience of many users and the potentially substandard image quality; these motivations have driven many research initiatives and commercial developments [160].

Table 3 provides an overview of currently available AI solutions in HHUSDs. Some HHUSDs use AI software programs that perform calculations, for example, to assess ejection fraction, cardiac output and bladder volume. Image recognition software automatically identifies and labels structures in B-mode US, and the image quality is continuously monitored.

Cardiology is the field most likely to have the most advanced AI integration, where commercialized Deep Learning (DL) AI tools are already guiding operators to achieve the correct field of view and heart visualization [161,162]. Large Language Models (LLMs) have also been adopted to help document and diagnose cardiac conditions, such as ventricular septal ruptures, by integrating keywords extracted from images with clinical history [163]. AI-assisted quantification of left ventricular function has already been implemented in several HHUSDs and has proven to be accurate [79,80,81,82,88,102,150,164] and cost-effective [102,165,166]. Researchers and startups have also developed interesting solutions to support the use of AI-driven POCUS across various gastroenterology and urology applications. AI solutions using traditional or advanced DL methods have been proposed to quantify liver fat specifically, as well as other liver etiologies, such as fibrosis and liver cancer [167,168,169]. Others have also proposed tackling problems in visualizing and characterizing several abdominopelvic organs, such as the pancreas, kidney, prostate, and bladder [161,163]. Finally, pulmonology is another field where DL approaches have contributed to solving both guidance and interpretation problems. Specifically, traditional DL methods are effective at quantifying B-lines from lung cine loops when used by less experienced users [170].

**Table 3:** Illustrative and non-exhaustive overview of artificial intelligence (AI)-based assistance reported or commercially available for HHUSDs (modified according to [171]).

Potentially Available AI-Tools *
**Workflow assistance**
AI-powered voice control of HHUSD functions
Automated image quality assessment
Automated probe position recognition
Capturing and real-time labelling of anatomical structures
eFAST assistance
AI-assisted examination report
**General Imaging**
Automated diameter measurements (vessel diameters, intima-media-thickness)
Automated volume calculations (e.g., bladder)
**Cardiovascular Imaging**
Inferior Caval Vein diameter/Collapsibility calculation
Left Ventricular Ejection Fraction calculation
Left-Ventricular Outflow Tract Velocity Index Time Integral calculation
Stroke volume, heart rate, cardiac output calculations
Identification and counting of B-lines of the lung
Doppler gate auto-localization and base-line adjustment
Automated Doppler-measurements
**Interventions**
Real-time needle-tracking and visibility enhancement

* Commercial availability, regulatory status, software versions, and individual functions vary between devices and geographical markets and may change rapidly.

Nonetheless, key limitations in the implementation, regulation, and adoption of AI-driven POCUS include:

1. obtaining adequate and well-curated data sets for training generalizable models, including strategies for prospective and retrospective data collection,
2. assessing algorithms through regulatory bodies and demonstrating robustness and true reliability of different algorithms across vendors, operators and/or acquisition parameters, in an efficient way, and
3. computational limits for more advanced models remain a challenge—training models can be very costly. In contrast, model deployment can be limited in terms of practical, real-time support when run on mobile devices.

Future exciting technical avenues for AI in POCUS (broadly applicable to US in general but particularly useful in POCUS) include advanced transformer-based models, access to raw, unprocessed US data, the potential for more advanced multi-parametric approaches, and the merging of robotics with POCUS via AI.

## 4. Discussion: Future Perspectives of HHUS

The great advantage of HHUSDs is their portability. HHUSDs may address currently unmet imaging needs in many areas. One possibility would be to send US images for supervision via telemedicine to centers that co-assess the HHUSD images. If required, more advanced imaging can be performed by experts using larger, more expensive equipment. It remains unclear whether the use of HHUSDs in this way has a beneficial impact on the medical treatment of patients, or whether it leads to an increased frequency of incidental sonographic findings, and consequently, of additional investigations [172,173].

Another significant advantage of HHUSDs is their relatively low purchase price compared with conventional cart-based US systems. This enables broad access to medical US diagnostics, especially in chronically underserved regions of the world with limited resources for medical equipment [174,175].

Given the current technical limitations of many HHUSDs, their clinical use should generally focus on applications for which the available device provides adequate image quality and functionality, even when examinations are performed by experienced operators. HHUS examinations may support bedside decision-making, streamline selected diagnostic pathways, and improve workflow efficiency when appropriately incorporated into routine clinical assessment and performed by clinicians with training tailored to the specific application [176]. Established specialist ultrasound services, including radiology- or department-based ultrasound facilities, remain essential for comprehensive or multiparametric ultrasound assessment and expert second-line evaluation [102,177,178]. Accordingly, investment in HHUS equipment should be accompanied by sustained support for qualified ultrasound services, training, quality assurance, and access to conventional and high-end ultrasound systems.

Effective implementation also requires secure transfer, storage, and documentation of HHUS examinations. Where appropriate and feasible, images and reports should be integrated into institutional electronic medical records and image-archiving systems.

Given the rapid market expansion and the relatively short operational lifespan of these devices [19], their environmental footprint warrants careful consideration. Most HHUSDs use piezoelectric transducer materials containing lead compounds, particularly lead zirconate titanate (PZT); without appropriate end-of-life management, they can contribute to electronic waste and pose environmental and occupational health risks.

These concerns may be especially relevant as HHUSD use extends beyond hospitals and larger health-care institutions to primary care, mobile and remote services, educational settings, and use by non-medical personnel [19], where access to formal collection and recycling pathways may be variable.

The magnitude of this burden has not yet been systematically quantified; life-cycle assessments addressing device longevity, material composition, repairability, reuse, and end-of-life pathways are therefore needed. Manufacturers and regulators should promote environmentally conscious design, material transparency, repairability, accessible take-back and recycling programs, and lower-impact transducer materials [34,40]. Manufacturers should operate environmental management systems, such as ISO 14001, and comply with applicable (Waste Electrical and Electronic Equipment) and RoHS (Restriction of Hazardous Substances) requirements. Embedding these principles throughout the HHUSD life cycle—from design and manufacturing through reuse, recycling, and disposal—would help ensure that expanding clinical and educational use remains both technologically innovative and environmentally responsible.

Despite recent advancements in HHUSD technology, miniaturization and ultra-portability is likely to impose limitations on hardware capabilities and post-processing algorithms. Advancements currently being pursued for HHUSDs include hardware improvement (e.g., new transducer technologies) and image enhancement technology, with refinements in beamforming technology (hybrid and completely digital beamforming; synthetic aperture techniques) and post-processing software [179,180]. However, these developments are currently limited by power consumption constraints and limited accessibility or raw radio-frequency signals. A promising approach to further improving image quality without hardware upgrades is the use of AI [181]. AI-based software may enhance the post-processing image quality of handheld ultrasound devices (HHUSDs) through neural network-based learning. In this approach, high-resolution ultrasound images are artificially degraded to approximate the lower image quality typically obtained with HHUSDs, thereby enabling the model to learn the inverse transformation when applied to HHUSD-acquired scans [182,183,184]. Building on this principle, a cycle-consistent generative adversarial network incorporating perceptual loss and trained on registered image pairs may improve native HHUSD images such that they more closely resemble images acquired with a more expensive and less portable high-end ultrasound system [185].

Cumulative advances in related fields—including semiconductor technology, increasing chip performance, novel battery technologies, improved low-heat circuitry and heat sink technology—are likely to narrow the technological gap between HHUSD and high-end ultrasound systems relatively quickly.

Wearables of all types have seen a marked increase in adoption in recent years, and viable medical use cases are increasingly emerging. Highly miniaturized ultrasound sensors and continued development of flexible ultrasound arrays—usable in larger or more anatomy conforming patches—are likely to have significant clinical impact within the coming years, building on current preclinical and early clinical work in this area [186,187,188].

Wearable and conformable ultrasound patches share with HHUS the technological trends toward miniaturization, semiconductor-based transducers, wireless connectivity, and increasingly automated image acquisition. However, their principal current use concept differs from HHUS: whereas HHUS is primarily intended for focused, time-limited clinician-performed assessment of a clinical question, wearable ultrasound is being developed predominantly for continuous or repeated monitoring of predefined anatomical or physiological targets [187,188]. These technologies should therefore currently be regarded as related but distinct approaches; in the future, however, technologies and applications may continue to converge. Future developments at the intersection of wearable, handheld, and high-end ultrasound devices will likely transform this distinction into a continuum of devices tailored to their respective clinical applications.

## 5. Limitations

This structured narrative technical review and international expert consensus has several limitations. The evidence base combined peer-reviewed studies with technical information from non-peer-reviewed sources and was not assessed using a systematic-review methodology or formal certainty-of-evidence grading. Comparative studies involved different HHUSDs, clinical applications, and predominantly subjective image-quality criteria, limiting direct comparisons. In addition, the rapidly evolving device market means that for some recently introduced technologies, technical specifications are available before prospective comparative clinical data. The purposive selection of the expert panel may have introduced selection effects. In addition, although conflicts of interest were disclosed, no formal conflict-of-interest-based restrictions or exclusions were applied during discussion or voting. Finally, the recommendations were developed by a selected international expert panel through three non-anonymous iterative discussion rounds followed by a single non-anonymous recommendation-specific vote rather than a formal Delphi process.

## 6. Conclusions

Handheld ultrasound technology has evolved from a niche innovation into a valuable tool for modern medical practice. Advances in miniaturization, digitization, and connectivity have enabled devices that can provide clinically adequate diagnostic imaging for an increasing range of focused applications while offering unprecedented portability and affordability. Contemporary HHUSDs now routinely include color and spectral Doppler and wireless functionality, with emerging integration of AI to support image acquisition, quantification, and interpretation.

The increasing availability of HHUSDs has the potential to expand access to diagnostic imaging and support timely bedside decision-making across diverse clinical settings, including resource-limited environments. Teleguidance, remote supervision, and appropriately validated AI-assisted applications may further extend the reach and functionality of HHUS, particularly where local ultrasound expertise is limited.

However, challenges remain. Regulatory frameworks and data security standards are still evolving. Appropriate user training and quality assurance must remain integral to the adoption process to ensure safe, reliable, and reproducible imaging outcomes.

Looking ahead, HHUS is likely to play an increasingly important role in point-of-care medicine, medical education, and telehealth. Its integration with AI and digital health networks will further expand its diagnostic and educational roles. To fully realize this potential, international collaboration among professional societies, regulators, and industry partners is needed to establish unified standards for training, quality assurance, and data management.

Ultimately, HHUS represents an important evolution in medical imaging that can expand access to focused ultrasound assessment at the point of care. Its clinical value depends on the diagnostic task, device capabilities, operator expertise, and clinical environment. HHUS should therefore complement rather than uniformly replace comprehensive multiparametric or high-end ultrasound examinations [4,10].

## 7. Key Messages and Recommendations

### 7.1. Standardization and Regulation

HHUSDs must be considered medical devices and comply with all regulations and standards for both medical devices and medical ultrasound machines, including safety, data privacy, information storage and documentation.

**Recommendation 1. The authors suggest developing and promoting international regulatory and quality frameworks for the appropriate and safe use of handheld ultrasound devices, including their integration with smart systems and the standardized documentation of handheld ultrasound examinations [agreement: 100%].**

### 7.2. Training and Competency

- Implement structured, competency-based training for all HHUSD users, including physicians, specialist nurses, midwives, paramedics, other medical professions, and students.
- Integrate supervision, continuous education, and quality assurance into clinical and telemedical practice.

**Recommendation 2. It is recommended that all users of handheld ultrasound should be offered a structured, competency-based training curriculum, which is tailored to the specific roles and responsibilities of each user group. Supervision by qualified personnel is required during the initial training phase and throughout continuing professional development. Clinical and telemedical practices involving handheld ultrasound must incorporate robust quality assurance processes [agreement: 100%].**

### 7.3. Quality Assurance and Data Security

- Ensure standardized documentation and integration of HHUS images into PACS and electronic health record systems.
- Apply robust cybersecurity measures, including encrypted data transfer, encrypted local storage, and multi-factor authentication.
- Prefer dedicated institutional smartphones or tablets whenever feasible; if personal devices are used, employ managed user accounts, automatic logout, and regular software updates.
- Use anonymized patient identifiers whenever appropriate and ensure compliance with applicable data protection legislation (e.g., GDPR, HIPAA, or equivalent national regulations).

**Recommendation 3. The storage of imaging data and reports from handheld ultrasound examinations should ensure auditability and comply with applicable data-protection regulations. Documentation should, whenever possible, be integrated into PACS and electronic health records. For smartphone- or tablet-based handheld ultrasound systems, dedicated institutional devices should be preferred. Where personal devices are used, managed institutional profiles or restricted user accounts, secure authentication, encrypted data storage and transfer, automatic logout, role-based access control, and institutional mobile-device management should be implemented to protect patient privacy and data security [agreement: 100%].**

### 7.4. Clinical Integration and Research

- Use HHUS as a complement, not a replacement, for comprehensive ultrasound examinations by experts.
- Encourage multicenter research to assess diagnostic performance, image quality, and clinical outcomes across devices, institutions, and practitioners.

**Recommendation 4. Image data from HHUS should be used, recognizing both its accuracy and its limitations, to support urgent decisions or emergency interventions at the point of care. The use of handheld ultrasound must not replace comprehensive, multiparametric ultrasound diagnostics or alternative expert imaging appropriate to the indication. The use of these imaging modalities must not be delayed if the required information cannot be obtained in sufficient quality with HHUS [agreement: 100%].**

### 7.5. Artificial Intelligence and Innovation

- Employ AI primarily to support guidance and quantification while maintaining human oversight and accountability.
- Promote transparency, validation, and monitoring of AI-driven diagnostic algorithms.

**Recommendation 5. Artificial intelligence can be used in handheld ultrasound to enhance image acquisition and interpretation, support workflow, and perform measurements. Manufacturers of handheld ultrasound devices must ensure strict validation of the clinical accuracy and safety of these AI applications and provide users with clear, accurate information about them. AI-supported handheld ultrasound should remain under appropriately trained clinical oversight, with accountability governed by applicable professional and regulatory frameworks [agreement: 100%].**

### 7.6. Environmental Sustainability

- Prioritize eco-friendly design, recycling systems, and take-back programs for HHUSDs.
- Support research into sustainable manufacturing practices.

**Recommendation 6. Manufacturers, regulatory authorities, and healthcare organizations should promote environmentally sustainable development of handheld ultrasound devices by supporting eco-conscious design, manufacturer-supported take-back and recycling programmes, responsible material management, and environmentally sustainable product life-cycle strategies [agreement: 100%].**

### 7.7. Global Health and Accessibility

- Expand HHUS training and device distribution to underserved and remote areas through international collaboration.
- Foster equitable access to diagnostic imaging by supporting sustainable, low-cost HHUS deployment worldwide.

**Recommendation 7. International and national ultrasound societies should promote handheld ultrasound training and sustainable device deployment to improve equitable access to diagnostic imaging in underserved and remote regions, in collaboration with international organizations committed to equitable global healthcare [agreement: 100%].**

## Figures and Tables

**Table 1:** Updated SWOT analysis for HHUSDs based on the EFSUMB Position Paper [16].

**Strengths**	**Weaknesses**
Portability: Small and lightweight (‘pocket sized’) Connectivity: Wired or wireless Wireless: transducer, power supply, charging, and/or data transfer Power: Battery operated or powered by connected device Rapid boot up Affordable Versatile: diagnostic and interventional utility Use in austere or remote settings (e.g., wilderness medicine, air travel) Entire device can be encased/bagged for infectious environments (e.g., Ebola or COVID-19) with ease of disinfection Interface with artificial intelligence and digital technology/applications Availability of ‘single probe’ technologies (“all-in-one”, “whole-body” probes)	Potentially limited screen size depending on configuration and capabilities Screen positioning during scanning Limited transducer–screen compatibility Image quality: generally limited Limited image settings or functionality Transducers: multiple transducers mostly required for different modalities Limited scanning time Variable recharging time Subscription fees for some systems Resistance to innovation Professional policy reservations (risk) Regulations and reimbursement not yet established in all countries Storage, loss of device Infection control issues Data integration into existing image documentation and archiving systems impossible or complicated with some systems
**Opportunities**	**Threats**
Applicability in low-resource health settings Increasing use and application across all subspecialties Increased adoption due to increasing availability and reduced costs Expedition of diagnoses in acute medicine or remote/community settings Telemedicine supervision (teleguidance). Remote access to AI or other off-device HHUS-software improvements	Equipment misplacement or theft Inadequate resolution for selected diagnostic indications, depending on the system Privacy issues (malware, third-party access) with smart-device-based HHUSD through web connectivity, teleguidance, and use of cloud storage Easy access for untrained users with risk for misuse/misinterpretation Lead exposure due to improper end-of-life disposal

**Table 2:** Summary of studies evaluating the performance of different HHUSDs for different clinical applications.

1st Author, Year	Study Design	Results
Le MPT, 2022 [22]	24 POCUS experts: three scans on the same standardized patients (Right upper quadrant view of the FAST protocol, transverse view of the neck, parasternal long-axis view of the heart) using 4 HHUSDs. Comparison in 3 categories of criteria: ease of use, image quality and overall operator satisfaction.	No single HHUSD was superior in all categories.
Merkel D, 2023 [23]	8 HHUSDs and a premium cart-based US system were used by 2 highly experienced examiners in a hospital department for internal medicine to answer 7 typical clinical questions (ascites, in-vivo needle tracking in ascites or pleural effusion, inferior vena cava diameter, in-vitro needle tracking in a gelatine phantom, gallbladder, focal liver lesion, pancreas), and video clips without patient data and system identification were recorded. The video clips were rated for B-scan quality and clinical relevance by 14 US experts.	For abdominal applications, relevant differences in B-scan quality were observed among HHUSDs from different manufacturers; the VScan Air (GE HealthCare) achieved a significantly higher average rating than the other HHUSDs in terms of both B-scan quality and clinical relevance.
Weimer JM, 2023 [24]	8 HHUSDs and a premium cart-based US system were used by 2 highly experienced examiners in an intensive care setting in 5 predefined clinical indications (inferior vena cava diameter, pleural effusion, pulmonary B-lines, gallbladder, and needle tracking in situ). Only the two best HHUSDs are mentioned in the study (Vscan Air, GE HealthCare; C3, Clarius). 16 intensive care US experts rated anonymized video clips for B-scan quality and clinical relevance.	In the intensive care setting, one HHUSD (VScan Air, GE HealthCare) performed significantly better than all other HHUSDs in terms of both B-scan quality and clinical relevance.
Merkel D, 2024 [26]	12 experienced examiners compared the performance of 9 HHUSDs on abdominal US in terms of B-scan quality, device handling, and software features on the same subjects.	Considerable variation of the B-scan quality of 9 HHUSDs, which may have implications for their clinical application. Vscan Air (GE HealthCare) and SonoEye (Chison, Jiangsu, China) achieved the best overall ratings with significant differences to the other 7 HHUSDs. The highest ranking was assigned to C3HD3 (Clarius) and Vscan Air for B-mode quality, to D5CL (SonoHealth, Guangzhou, China) for device handling and to SonoEye (Chison) and Vscan Air for software.
Perez-Sanchez A, 2024 [27]	35 POCUS experts of different specialties: 3 scans in random order on the same standardized patients (RUQ abdominal scan, cardiac 4-chamber view, combined superficial neck and lung scan) comparing 6 HHUSDs. Comparison of image quality, overall ease of use, and overall operator satisfaction.	All 6 HHUSDs had notable advantages and disadvantages, with no single HHUSD demonstrating superior image quality for all scans. VScan Air (GE Healthcare) was rated highest for overall image quality, overall ease of use and was the most preferred HHUSD for purchase by POCUS experts.
Leung SK, 2025 [28]	8 pediatric POCUS experts: 3 scans in random order in a single pediatric model (RUQ abdominal scan, cardiac 4-chamber view, combined superficial neck and lung scan) comparing 6 HHUSDs. Comparison of image quality, overall ease of use, and overall operator satisfaction.	No device was clearly superior across all applications, and all 6 HHUSDs had important advantages and disadvantages.
Naas A, 2026 [29]	Rating of 180 videoclips from 10 healthy individuals with scans of 3 predefined abdominal regions (pancreas, liver hilum, sigmoid colon) using 5 HHUSDs and one cart-based high-end US system by 22 US experts in a blinded setup. Comparison (0–10 Likert Scale) of overall image quality, spatial resolution, dynamic range, contrast, depth penetration.	Image quality of all 5 HHUSDs was rated inferior to the high-end US device with significant differences between the 5 HHUSDs. C3 (Clarius) was rated best, followed by Lumify (Philips) and Vscan Air (GE HealthCare). Butterfly’s iQ+ was rated worst. Overall impression of image quality correlated with the individual quality parameters without superiority of any specific quality parameter. Image quality ratings were impacted by the device used by the US experts in daily practice, but hardly by their US experience.

## Data Availability

No new data were created or analyzed in this study. Data sharing is not applicable to this article.

## Abbreviations

The following abbreviations are used in this manuscript:

AI	Artificial Intelligence
cMUT	capacitive Micromachined Ultrasonic Transducer
COVID	Coronavirus Disease
DICOM	Digital Imaging and Communications in Medicine
DL	Deep Learning
EFSUMB	European Federation of Societies for Ultrasound in Medicine and Biology
EHR	Electronic Health Record
ESR	European Society for Radiology
FAST	Focused Assessment by Sonography in Trauma
GDPR	General Data Protection Regulation
HHUS	Handheld Ultrasound
HHUSD	Handheld Ultrasound Device
HIPAA	Health Insurance Portability and Accountability Act
LLM	Large Language Models
NISV	Ordinance on Protection against Harmful Effects of Non-Ionizing Radiation in Human Applications
PACS	Picture Archiving and Communication System
pMUT	Piezoelectric micromachined ultrasonic transducer
POCUS	Point-of-Care Ultrasound
ROHS	Restriction of Hazardous Substances
RUQ	Right upper quadrant (of the abdomen)
SWOT	Strengths, Weaknesses, Opportunities, and Threats
US	Ultrasound
WEEE	Waste Electrical and Electronic Equipment
WFUMB	World Federation for Ultrasound in Medicine and Biology
